# Knockdown of Gastrin Promotes Apoptosis of Gastric Cancer Cells by Decreasing ROS Generation

**DOI:** 10.1155/2021/5590037

**Published:** 2021-04-14

**Authors:** Yan Liu, Jihai Zhu, Jun Liu, Xueman Ma, Jun Zhao, Zhanhai Su

**Affiliations:** ^1^Immunology Department, School of Medicine, Qinghai University, Qinghai 810000, China; ^2^Cardiovascular Surgery Department, The Affiliated Hospital of Qinghai University, Qinghai 810000, China

## Abstract

Overexpressed gastrin is reported to promote oncogenesis and development of gastric cancer by inhibiting apoptosis of cancer cells; however, the underlying mechanism remains unclear. Our study is aimed at revealing the mechanism underlying the effect of gastrin on apoptosis of gastric cancer cells. Gastrin-interfering cell line was constructed by stably transfecting gastrin-specific pshRNA plasmid to gastric cancer cell line BGC-823. Then, differentially expressed proteins between untreated BGC-823 and gastrin-interfering BGC-823 cell lines were detected by the iTRAQ technique. GO and KEGG analysis was used to analyze the differentially expressed genes that code these differentially expressed proteins. The Annexin V-FITC staining assay was used to detect gastric cancer cell apoptosis. The DCFH-DA fluorescent probe staining assay was used to measure intracellular ROS. Mitochondrial membrane potential was detected by flow cytometry. Western blot was used to analyze the mitochondria respiratory chain proteins and apoptosis-related proteins. A total of 107 differentially expressed proteins were identified by iTRAQ. GO and KEGG analysis showed that proteins coded by the corresponding differentially expressed genes were mainly enriched in the mitochondrial oxidative respiratory chain, and the expression of three proteins (COX17, COX5B, ATP5J) was upregulated. The three proteins with higher scores were verified by Western blot. The apoptosis rate of the gastrin knockdown cancer cell was significantly increased; meanwhile, gastrin knockdown leads to increase of membrane potential and decrease of intracellular ROS production. Additionally, Bax was significantly increased, whereas NF-*κ*B-p65 and Bcl-2 were downregulated after knockdown of gastrin. Concomitantly, pretreatment with NAC reversed the effect of gastrin on the Bax and Bcl-2 expression. Gastrin promotes the production of ROS from mitochondria, activates NF-*κ*B, and inhibits apoptosis via modulating the expression level of Bcl-2 and Bax.

## 1. Introduction

Gastrin is an important polypeptide hormone synthesized by gastroduodenal G cells and contributes to the regulation of a variety of gastrointestinal tract functions, including acid secretion, motility, and epithelial proliferation [1]. Despite the known role of gastrin as a normal gastrointestinal hormone, an increasing number of studies have shown its “dark side” [2–10]. Gastrin has been reported to be abnormally expressed in a variety of tumors, such as pancreatic cancer [3], colorectal cancer [4], lung cancer [5], and gastric cancer [6], and it can promote proliferation and metastasis of tumors [7], alter the immune cell signature in the tumor microenvironment [8], and inhibit cancer cell apoptosis [9, 10]. To date, the role of gastrin in inhibiting apoptosis of gastric cancer cells has been fully elucidated [11, 12]; however, the underlying mechanism remains to be further investigated.

Generally, mitochondria not only generate energy for the vital activities of eukaryotic cells but also participate in many biological processes, such as energy metabolism [13], apoptosis [14], ROS production [15], and calcium homeostasis [16, 17]. Studies have shown that the abnormal expression of mitochondrial respiratory chain proteins is closely related to a variety of diseases, especially tumors [18, 19]. In the 1930s, Otto Warburg suggested that “respiration damage” was a pivotal feature of cancer cells [20, 21]. Subsequently, a large number of studies have shown a decrease of mitochondrial respiration and oxidative phosphorylation in cancer [22, 23]. Today, the concept that mitochondrial dysfunction is one of the most prominent features of cancer cells has been widely accepted.

Mitochondria are the major source of cellular reactive oxygen species (ROS). In some cases, the inhibition of respiratory activity clearly leads to enhanced generation of ROS [22]. By acting both as mutagens and cellular mitogens, excessive ROS changes the cellular redox status and oxidative stress [23] and affects the activities of sensitive transcription factors to regulate the gene expression and promote cancer cell proliferation [24, 25]. Thus, when mitochondria malfunction, they produce excessive ROS, which may be involved in carcinogenesis of cancer.

Whether gastrin could affect the production of ROS by modulating the oxidative respiratory chain of mitochondria in gastric cancer cells and thus affect cancer cell apoptosis are unclear. Herein, we aim to address these questions. Our findings can provide experimental evidence and lay the foundation for further understanding the mechanism underlying the antiapoptosis effect of gastrin in gastric cancer cells.

## 2. Materials and Methods

### 2.1. Cancer Cell Lines and Cell Treatment

The human gastric cancer cell line BGC823 (a gift from Laboratory of Molecular Oncology, Peking University Cancer Hospital) was used in this study. Cells were cultured with DMEM complete medium (Gibco, Grand Island, NY, USA), which contained 5% fetal bovine serum (FBS, Gibco, Grand Island, NY, USA), penicillin (100 U/ml), and streptomycin (100 mg/ml). Cells were incubated at 37°C with 5% CO_2_. NAC (N-acetyl-L-cysteine) (Beyotime, Shanghai, China; 10 mM), the inhibitor of ROS, was added to the cells and incubated for 4 h. Then, cells were collected for further analysis.

### 2.2. Construction of Plasmid and Transfection

For RNA interference, the chemically synthesized annealed siRNA duplexes were inserted between the BamHI and HindIII sites on the Psilencer™3.1–H1 plasmid (Ambion, Austin, TX, USA) to generate gastrin-specific-interfering short hair RNA (shRNA) plasmid that was named as pshRNA plasmid, while the PsilencerTM3.1–H1 plasmid without siRNA insertion was used as empty plasmid. Cells were cultured to 60–70% confluence in 35 mm plates and then transfected with recombinant plasmids using the Lipofectamine2000 reagent (Invitrogen, Carlsbad, CA, USA) according to the manufacturer's instruction. Selective medium containing 400 g/ml G418 was used to screen stable transfected cell clones after transfecting for 48 h. The target nucleotide sequences that can interfer with the gastrin gene expression were as follows: AAGAAGAAGAAGCCTATGGAT; the sequences of shRNA designed based on the target nucleotide sequences were mentioned above: F:5′-ATCC GAAGAAGAAGCCTATGGAT TTCAAGAGA ATCCATAGGCTTCTTCTTCTT TTTTGGAAA-3′; R:5′-AGCTTTTCCAAAA AAGAAGAAGAAGCCTATGGAT TCTCTTGAA ATCCATAGGCTTCTTCTTC G-3′.

### 2.3. Real-Time Quantitative PCR

Total RNA was extracted from culture cells using TRIzol Reagent (Invitrogen, Carlsbad, CA, USA). Total RNA (5 *μ*g) was reverse transcribed into cDNA with the SuperScript III First-Strand Synthesis System (Invitrogen, Carlsbad, CA, USA). The primer sequences were as follows: gastrin, 5′GAC GAG ATG CAG CGA CTA TGT 3′ (sense) and 5′GGG TCT GCC ACG AGG TGT 3′ (antisense) and *β*-actin, 5′CGG GAA ATC GT GCG TGA CAT T 3′ (sense) and 5′CTA GAA GCA TTT GCG GTG GAC 3′ (antisense). Real-time quantitative PCR was performed using ABI PRISM7700 Sequence Detection (Applied Biosystems, Foster City, CA, USA). *β*-Actin was used as an internal control. PCR reaction procedure was 28 cycles of 94°C denaturation for 30s, 62°C annealing for 30 s, and 72°C elongation for 30 s and a final elongation at 72°C for 10 min. The relative mRNA expression levels of gastrin were calculated using the 2^-△△Ct^ method.

### 2.4. iTRAQ and Bioinformatic Analysis

Stable cell lines with gastrin knockdown were constructed in BGC823 cells via interfering with pshRNA (BGC823-Gastrin KD), and cell lines without gastrin knockdown were constructed in BGC823 cells via interfering with empty plasmid (BGC823-Ctrl cell). BGC823-Gastrin KD and BGC823-Ctrl cells were lysed in lysis buffer (50 mmol/L Tris–Cl (pH 6.8), 100 mmol/L DTT, 2% SDS, 10% glycerol). Equal amounts of protein (50 *μ*g) were sent to Beijing Huada Protein Research & Development Center for iTRAQ (isobaric tags for relative and absolute quantitation) analysis. The differentially expressed proteins were identified by iTRAQ. The corresponding genes that code these differentially expressed proteins were subjected to GO and KEGG analysis using the advantage *R* software.

### 2.5. Mitochondrial Membrane Potential Detection

Mitochondrial membrane potential was detected by fluorescence microscopy and flow cytometry using 5,5′,6,6′-tetrachloro-1,1′,3,3′-tetraethylbenzimidazolylcarbocyanine iodide (JC-1; Beyotime, Shanghai, China). BGC823-Gastrin KD and BGC823-Ctrl cells were cultured for 24 h and then incubated with 10 *μ*M JC-1 for 2 h in a CO_2_ incubator at 37°C. Then, cells were washed twice with PBS. Green monomers (ex485nm/em535nm) and red J-aggregates (ex560nm/em595nm, a sensitive marker of *Δψ*m) were detected by laser confocal scanning microscope (TCS- SP5; Leica, Mannheim, Germany) and by flow cytometry, respectively (BD FACSAria, San Jose, CA, USA).

### 2.6. Dichloro-Dihydro-Fluorescein Diacetate (DCFH-DA) Assay

ROS level in gastric cancer cells was detected by the ROS detection kit (Beyotime Biotechnology, Shanghai, China). The DCFH-DA probe was diluted with a serum-free medium at 1 : 1000, and the work concentration was 10 *μ*M. After removal of culture medium, 1 ml of diluted DCFH-DA probe was added to cells and incubated at 37°C for 20 min. Later, cells were washed three times with serum-free medium to completely remove DCFH-DA probe that did not enter the cells and then observed by laser confocal scanning microscope (TCS- SP5; Leica, Mannheim, Germany) and analyzed by flow cytometry (BD FACSAria, San Jose, CA, USA).

### 2.7. Flow Cytometry for Analysis of Apoptosis

To detect the apoptosis of cells, Annexin V-FITC apoptosis detection kit (Beijing Baosai Biotech, Beijing, China) was used. Briefly, cells were collected and suspended in prechilled PBS. After three times washing with PBS, cells were resuspended in 200 *μ*l binding buffer with 10 *μ*l Annexin V-FITC and incubated in dark for 15 min at room temperature. Later, 300 *μ*l binding buffer with 5 *μ*l PI solution was added into the cell suspension. Then, the apoptotic rate of gastric cancer cells was detected by flow cytometer (BD FACSAria, San Jose, CA, USA) within an hour.

### 2.8. Western Blot Analysis

Two stable clones of BGC823-Gastrin KD cells were selected and, respectively, named as PshrNa-A1and PshrNa-B3. BGC823-Ctrl cells and untreated BGC823 cells were taken as control. These cells were subjected to lysis (50 mmol/L Tris–Cl (pH 6.8), 100 mmol/L DTT, 2% SDS, and 10% glycerol). Equal amounts of protein (50 *μ*g) were then separated on 12% SDS–PAGE and transferred to PVDF membrane (Pharmacia, (GE), USA). The membrane was blocked with 5% nonfat milk for 1 h and then incubated with the specific antibody: anti-gastrin (Abcam, Cambridge, MA, USA) (1 : 500), anti-I*κ*B-*α* (Santa Cruz, CA, USA) (1 : 500), anti-p-I*κ*B-*α* (Santa Cruz, CA, USA) (1 : 500), anti-NF-*κ*B (Santa Cruz, CA, USA) (1 : 500), anti-Bcl-2 (US Biological, Boston, USA) (1 : 500), anti-Bax (Epitomics, Burlingame, CA, USA) (1 : 500), anti-COX17(Abcam, Cambridge, MA, USA) (1 : 500), anti-COX5B(Abcam, Cambridge, MA, USA) (1 : 500), anti-ATP5J(Abcam, Cambridge, MA, USA) (1 : 500), and anti-*β*-actin (Santa Cruz, CA, USA) (1 : 20000) overnight at 4°C. Horseradish peroxidase-conjugated secondary antibodies (Santa Cruz, CA, USA) were used at a 1 : 2000 concentration. Color development was performed with the ECL system (GE Health Care, Little Chalfont, Buckinghamshire, UK).

### 2.9. Statistical Analysis

Data was analyzed by using statistical software SPSS19.0 (IBM, Armonk, NY, USA). The chi-square test was used for counting data comparison. Differences between two groups were evaluated using two-sided unpaired Student's *t*-test. Comparison of more than two groups was performed with one-way ANOVA followed by Tukey's multiple comparison test. Statistical significance was set at the level of *P* < 0.05.

## 3. Results

### 3.1. The pshRNA Effectively and Persistently Suppresses the Gastrin Expression in BGC823 Cells

**Figure 1:**
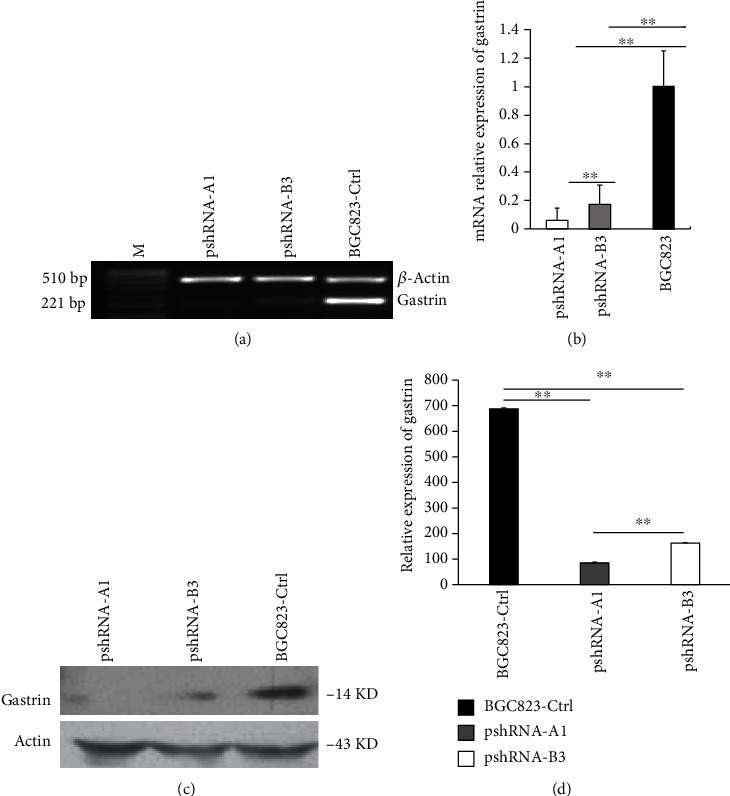
Knockdown of the gastrin expression in BGC823 cell line by RNA interference. (a) RT-PCR analysis detected more obvious interfering effect on gastrin in cell clones pshRNA-A1 and pshRNA-B3. (b) Level of gastrin mRNA was measured by real-time RT-PCR. The error bars represent the standard deviations of triplicate experiments, ^∗∗^*P* < 0.001. (c) Level of gastrin protein in clones pshRNA-A1, pshRNA-B3, and BGC823-Ctrl by Western blotting. (d) Grayscale scanning results of Western blot, ^∗∗^*P* < 0.001.

We successfully constructed pshRNA recombinant plasmids targeting gastrin. After 22 days of selection, positive cell clone was obtained. To examine whether shRNA induces gene silencing, qRT-PCR and Western blot were performed to detect the gastrin expression. We found that the gastrin mRNA expression was significantly downregulated in two clones of pshRNA-A1 and pshRNA-B3 (Figures 1(a) and 1(b)). Western blot analysis showed the dramatic decrease of gastrin protein levels in two clones compared to control cells BGC823-Ctrl (Figures 1(c) and 1(d)). Because the downregulation of the mRNA and protein expression level of gastrin was more significantly in pshRNA-A1 than that in pshRNA-B3, we selected the clone with stable pshRNA-A1 transfection to represent BGC823-gastrin KD for follow-up experiments.

### 3.2. Knockdown of Gastrin Results in Expression Level Changes of Proteins Involved in the Oxidative Phosphorylation Function of Cell Mitochondria in Gastric Cancer Cells

**Figure 2:**
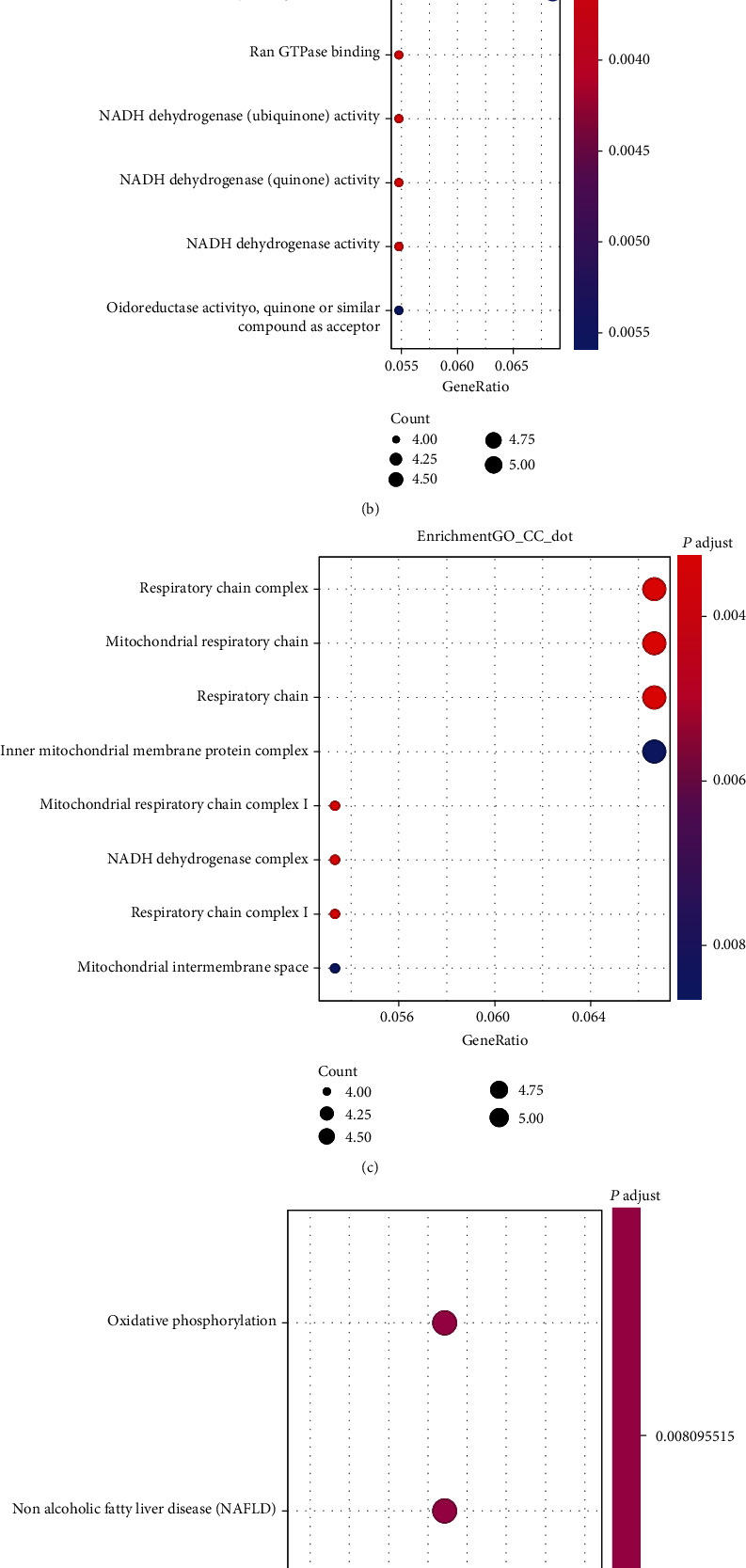
Knockdown of gastrin results in mitochondrial dysfunction in gastric cancer cells. Enrichment analysis of the differentially expressed genes identified by iTRAQ in gastrin knockdown gastric cancer cells. The bubble diagrams display the enrichment results of the 107 genes differentially expressed in gastrin knockdown gastric cancer cells compared with control cells. (a) Biological processes. (b) Molecular functions. (c) Cellular components. (d) KEGG pathway analysis. (e) Western blot was used to detect the expression of mitochondrial-related proteins (COX17, COX5B, and ATP5J) in BGC823 cells after interfering with gastrin, ^∗∗^*P* < 0.001 vs BGC823-Ctrl.

iTRAQ analysis is one of the mass spectrometry-driven protein quantification methods and always applied to identify differentially expressed proteins between groups in a high throughput manner. Here, iTRAQ analysis was used to identify the differentially expressed proteins between BGC823-Gastrin KD and BGC823-Ctrl cells. We found 107 significantly differently expressed proteins (either overexpression or downexpression) when gastrin was knocked out. GO analysis showed that these differentially expressed proteins mainly located in respiratory chain complex (Figures 2(a)–2(c)), where they participate primarily in mitochondrial respiratory chain complex assembly. They also had the oxidoreductase activity. KEGG pathway analysis showed that these proteins were enriched in oxidative phosphorylation pathways (Figure 2(d)).

Western blots were performed for the proteins of COX17, COX5B, and ATP5J, which are important components of mitochondrial oxidation respiratory chain, to confirm iTRAQ results. As shown in Figure 2(e), COX17, COX5B, and ATP5J were overexpressed after gastrin knockdown in gastric cancer (*P* < 0.01). These results indicate for the first time that the knockdown of gastrin in gastric cancer cells may affect the oxidative phosphorylation function of cell mitochondria via upregulating the COX17, COX5B, and ATP5J expression level.

### 3.3. Knockdown of Gastrin Affects Mitochondrial Membrane Potential

**Figure 3:**
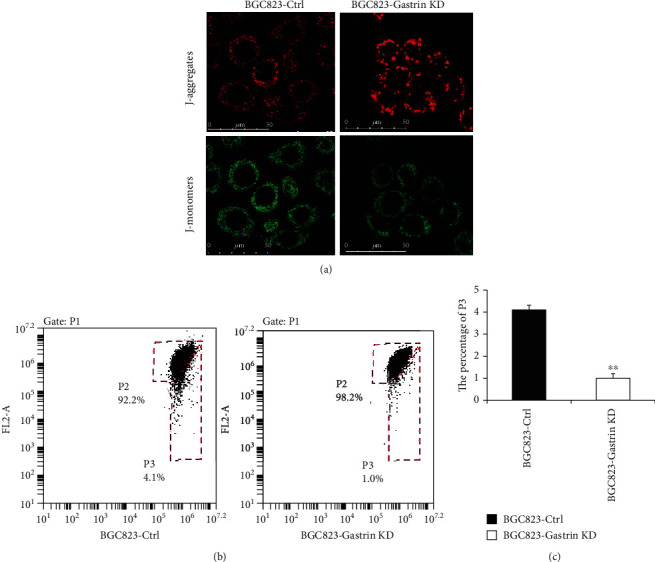
Knockdown of gastrin affects the mitochondrial membrane potential. (a) JC-1 staining was detected cell mitochondrial membrane potential and analyzed by laser-scanning confocal microscopy. The images showed representative fluorescence micrographs of JC-1-stained gastrin KD cells and control cells. (b) Mitochondrial membrane potential evaluated by JC-1 staining and analyzed by flow cytometry. Cell populations with higher and lower JC-1 aggregated staining are marked with high percentage of p3 and low percentage of p3, respectively. A decrease in the p3 indicates a rise in mitochondrial membrane potential. Each column represents the mean ± SD of three independent experiments (^∗∗^*P* < 0.01 vs BGC823-Ctrl).

Since the mitochondrial associated protein was overexpressed in gastrin-silenced cells, we hypothesize that there may be some mitochondrial defects in cancer cells. So, we used JC-1 staining to detect mitochondrial membrane potential. As shown in Figure 3(a), in BGC823-Ctrl cells, the green fluorescence was stronger, and the red fluorescence was weaker. However, after interfering with the gastrin expression, the red light fluorescence enhanced, and the green fluorescence was weakened in BGC823-Gasrin KD cells. Consistently, flow cytometry showed increased red fluorescence intensity, decreased green fluorescence intensity, and increased red/green fluorescence intensity ratio in BGC823-Gasrin KD cells (Figures 3(b) and 3(c), *P* < 0.01). These results indicate that gastrin knockdown increases mitochondrial membrane potential in gastric cancer cells.

### 3.4. Knockdown of Gastrin Promotes Apoptosis of Gastric Cancer Cells

**Figure 4:**
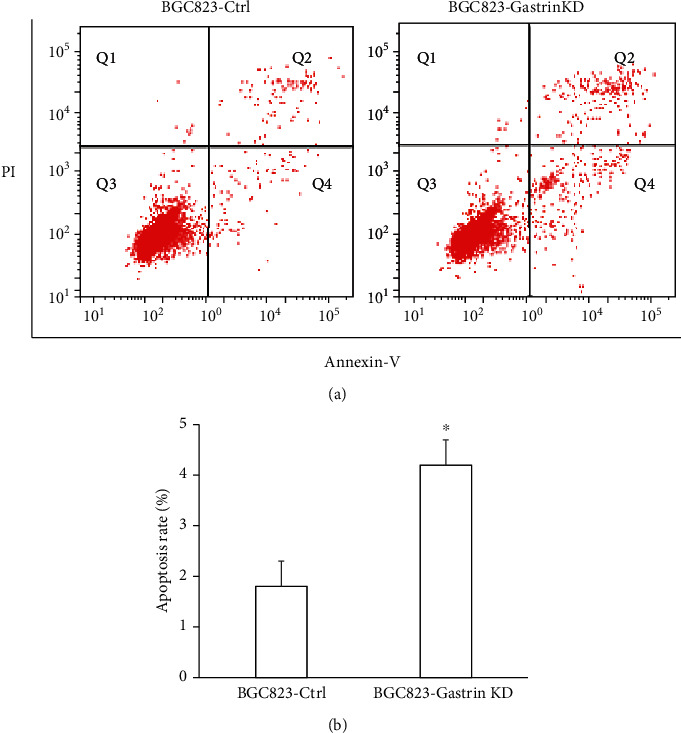
Knockdown of gastrin promotes apoptosis of gastric cancer cells. (a) The apoptosis was detected by flow cytometry. (b) The apoptotic rate of gastric cancer was comparatively analyzed (^∗^*P* < 0.05 vs BGC823-Ctrl).

Mitochondria are important organelles for regulating cell apoptosis. To evaluate the effect of gastrin knockdown on apoptosis of gastric cancer cells, the Annexin V-FITC apoptosis assay was done. Results showed that the apoptotic rate of gastric cancer cells was significantly higher in BGC823-Gasrin KD cells than that in BGC823-Ctrl cells, which were 4.2% and 1.8%, respectively (Figures 4(a) and 4(b), *P* < 0.05). This data indicates that knockdown of gastrin significantly promoted apoptosis of gastric cancer cells.

### 3.5. Knockdown of Gastrin Reduces the Level of Mitochondrial ROS in Gastric Cancer Cells

**Figure 5:**
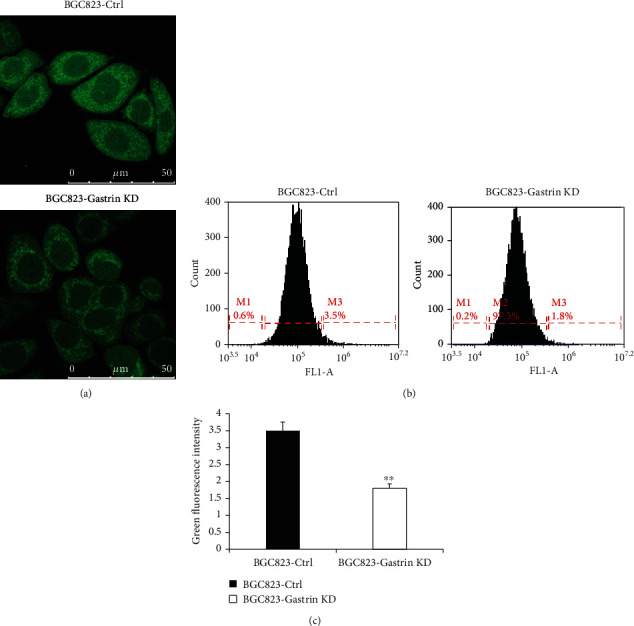
Knockdown of gastrin inhibits ROS production in gastric cancer cells. (a) Representative photos of the DCFH-DA probe assay under laser-scanning confocal microscopy (scale bar 50 *μ*M). (b) Flow cytometry was performed to detect the ROS level using DCFH-DA molecular probe. (c) Statistical results of the ROS level. Data are expressed as means ± SD (^∗∗^*P* < 0.01 vs BGC823-Ctrl).

As known, intracellular ROS is mainly generated in mitochondria, and thus we speculate that gastrin knockdown may affect the ROS generation. To verify this speculation, the ROS level in mitochondria of gastric cancer cells was detected by DCFH-DA probe. The green fluorescence intensity is proportional to the level of intracellular ROS. Both of confocal microscope (Figure 5(a)) and flow cytometry (Figures 5(b) and 5(c)) results showed that green fluorescence intensity was significantly decreased after knockdown of gastrin in BGC823 (*P* < 0.01), indicating that intracellular ROS production decreased after knockdown of the gastrin expression.

### 3.6. Knockdown of Gastrin Affects the Expression of Mitochondrial Apoptotic-Related Proteins in Gastric Cancer Cells

**Figure 6:**
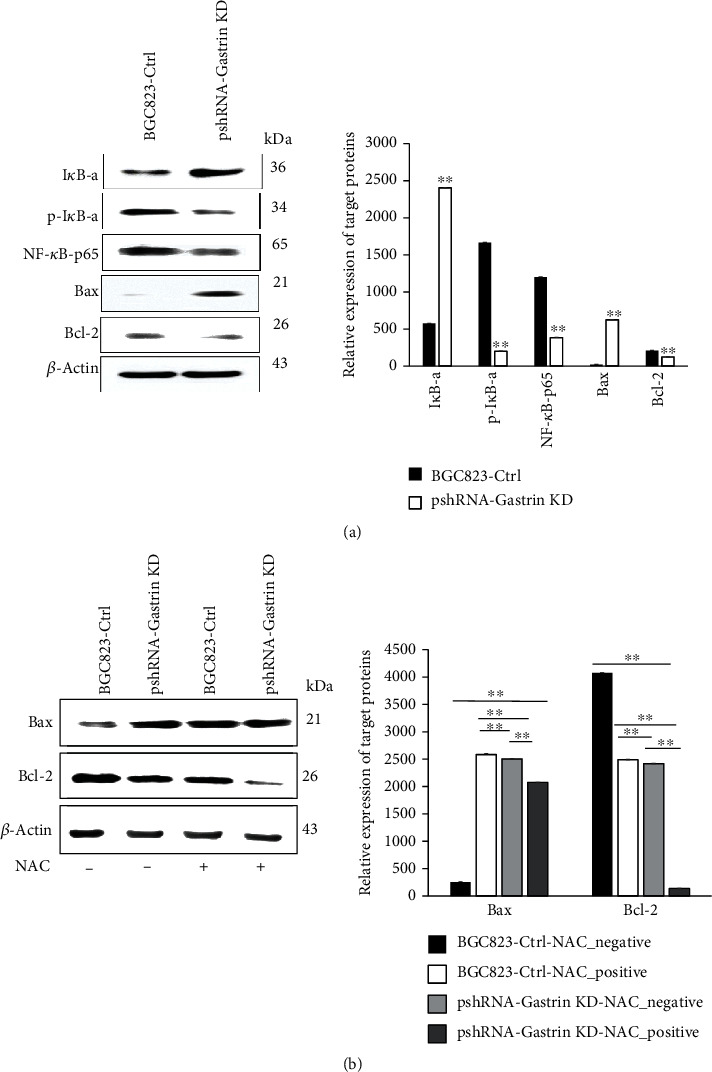
Knockdown of gastrin inhibits the expression of mitochondrial apoptotic-related protein in gastric cancer cells. (a) Western blot analysis of NF-*κ*B and Bcl-2 family proteins in gastrin KD cells compared with control cells. *β*-Actin was used as a loading control. Data are expressed as means ± SD (^∗∗^*P* < 0.01). (b) Western blot analysis of Bcl-2 family proteins in gastrin KD cells compared with control cells after pretreatment with NAC. Data are expressed as means ± SD (^∗∗^*P* < 0.01).

Western blot was performed to determine the effect of gastrin knockdown on protein expressions of I*κ*B-*α*, p-I*κ*B-*α*, NF-*κ*B-p65, Bax, and Bcl-2. The results showed that the expression of I*κ*B-*α* was significantly upregulated, while p-I*κ*B-*α* and NF-*κ*B-p65 were significantly downregulated after knockdown of gastrin in gastric cancer cells (Figure 6(a)) (*P* < 0.05). These data suggest that gastrin can promote THE NF-*κ*B activity.

Since both expression levels of Bax and Bcl-2 proteins are regulated by NF-*κ*B [26], we used Western blot analysis to test whether gastrin-induced NF-*κ*B activation affects the Bax and Bcl-2 expression. Our results showed that gastrin knockdown caused reduction of Bcl-2 levels and elevation of Bax levels (Figure 6(a)).

To further confirm whether the above protein expression change induced by gastrin is associated with ROS generation, we pretreated cells with the antioxidant NAC, which is a ROS scavenger. Compared with untreated BGC823-Ctrl cells, the Bax expression level was upregulated, and the Bcl-2 expression level was downregulated in BGC823-Ctrl cells pretreated with NAC. Moreover, Bax and Bcl-2, respectively, had similar expression levels between BGC823-Gasrin KD cells and GC823-Ctrl cells treated with NAC (Figure 6(b), *P* < 0.01), indicating that gastrin-induced Bax and Bcl-2 expression changes are ROS-dependent.

## 4. Discussion

In this study, we found that knockdown of gastrin in gastric cancer cells could result in the overexpression of COX17, COX5B, and ATP5J proteins, elevation of mitochondrial membrane potential, and reduction of ROS production in gastric cancer cells. Moreover, our results also showed that gastrin knockdown caused reduction of Bcl-2 levels and elevation of Bax levels in a ROS-dependent manner, indicating that gastrin knockdown promotes gastric cancer cell apoptosis by modulating the expression levels of Bax and Bcl-2.

Gastrin is a traditional oncogenic factor. It could promote angiogenesis by activating HIF-1*α*/*β*-catenin/VEGF signaling in gastric cancer [27]. In addition, it is proved that gastrin functions as a stimulator of the metastasis of gastric carcinoma through the *β*-catenin-TCF4 pathway [28]. Moreover, gastrin could regulate autophagy of gastric cancer cells through the AMPK*α*/Ras/Raf/MEK/ERK pathway and plays a procancer role [29]. Although its role as an oncogenic factor has been very clear, there has been no new breakthrough in recent years. To further characterize the function of gastrin in GC development, we investigated protein expression changes by performing iTRAQ analysis. We unexpectedly detected that knockdown of gastrin resulted in the overexpression of several mitochondrial respiratory chain-related proteins and elevated mitochondrial membrane potential in gastric cancer cells.

Recently, it is reported that there is a close relationship between mitochondria and tumor development [26, 30]. Mitochondrial dysfunction, especially the abnormal expression of mitochondrial respiratory chain-related proteins, is reported to be closely related to oncogenesis, development, invasion, and metastasis of various tumors [31, 32]. For example, it is reported that the expression level of NDUFA13, a subunit of mitochondrial respiratory chain complex I, decreases in a variety of tumors [33], such as lung [34], gastric [35], and liver [36] tumors, which promotes tumorigenesis by inhibiting tumor cell apoptosis [37]. Here, we found the effect of gastrin on the expression levels of COX17, COX5B, and ATP5J proteins that are involved in the mitochondrial respiratory chain, which may reveal one of the important mechanisms of gastrin as a cancer promoting factor.

ROS is the product of normal oxidative metabolism of cells, and endogenous ROS are mainly produced by mitochondria [38]. Dysfunction of the mitochondrial electron transport chain (such as blocking the function of complexIor complex II) significantly promoted ROS generation [39], resulting in accumulation of ROS in mitochondria and cytoplasm, leading to oxidative damage and abnormal cell function. Under hypoxia condition, the expression of NDUFA5, NDUFS6, NDUFA9 (mitochondrial respiratory chain complex І), cytochrome B (complex III), mitochondrial-encoded subunit I (COXI), and nuclear-encoded subunit IV (COXIV) (complex IV) decreased in TE-1 cells (Esophageal squamous cell carcinoma cell line), which promoted ROS generation and further accelerated the proliferation of tumor cells [40]. We, thus, propose that gastrin is the initial/critical event that results in mitochondria ROS elevation and affects cell apoptosis. As expected, our data demonstrate that BGC823-Gastrin KD cell, which had mitochondrial dysfunction, resulted in reduction of ROS production via upregulating COX17, COX5B, and ATP5J expression and increasing mitochondria potential, suggesting that gastrin is one of key factors that influence the ROS level.

Compelling evidence suggests that the increased ROS stress in cancer cells has a pivotal role in the acquisition of the hallmarks of cancer, including disruption of cell death signaling [41], since NF-*κ*B is a redox-sensitive transcription factor that is activated by increased levels of ROS [42]. And NF-*κ*B also has been shown to induce the expression of some members of the antiapoptotic proteins [43]. Thus, we examined whether knockdown of gastrin is associated with deactivation of NF-*κ*B in a ROS-dependent manner in gastric cancer cells. Our data showed that gastrin knockdown upregulated the protein expression level of I*κ*B-a and downregulated NF-*κ*B activation in GC cells depending on low levels of ROS. Thus, NF-*κ*B pathways may be involved in the gastrin mediated regulation of apoptosis. We also observed a decrease in the expression of antiapoptotic Bcl-2 proteins and an increase in proapoptotic Bax in gastrin knockdown cells. Furthermore, compared with untreated BGC823-Ctrl cells, the Bax expression level was upregulated, and Bcl-2 expression level was downregulated in BGC823-Ctrl cells pretreated with NAC. Moreover, Bax and Bcl-2 had similar expression level changes between BGC823-Gasrin KD cells and GC823-Ctrl cells treated with NAC, indicating that gastrin-induced Bax and Bcl-2 expression changes are ROS-dependent, and gastrin knockdown may promote apoptosis via upregulating Bax and downregulating Bcl-2.

## 5. Conclusions

To sum up, gastrin knockdown upregulates the expression of mitochondrial respiratory chain proteins, including COX17, COX5B, and ATP5J; increases the mitochondrial potential; and decreases the production of ROS. Moreover, knockdown of gastrin is associated with deactivation of NF-*κ*B in a ROS-dependent manner in gastric cancer cells and upregulation of Bax and downregulation of Bcl-2, thus promoting apoptosis.

## Data Availability

The data are available from the corresponding author.
